# Factors Associated with Mental Health Outcomes and the Level of Work Engagement Among Health Care Workers Exposed to Coronavirus Disease 2019 in Tunisia

**DOI:** 10.1192/j.eurpsy.2022.1305

**Published:** 2022-09-01

**Authors:** I. Betbout, B. Amemou, A. Ben Haouala, Y. Touati, M. Benzarti, F. Zaafrane, A. Mhalla, L. Gaha

**Affiliations:** 1 fattouma bourguiba hospital, Psychiatry, Psychiatry Research Laboratory Lr05es10 “vulnerability To Psychoses” Faculty Of Medicine Of Monastir, University Of Monastir, monastir, Tunisia; 2 University Hospital of Sahloul and Farhat Hached, Emergency, sousse, Tunisia

**Keywords:** Covid19 pandemic-mental health-health care workers-depressioninsomnia-distress-anxiety-work engagem

## Abstract

**Introduction:**

Heath workers especiallyin the emergency rooms and emergency medical services are exposed to sustained stress which had increased due to the Pandemic situation

**Objectives:**

To search for factors associated with mental disorders among health workers during the Covid 19 pandemic

**Methods:**

Data were collected through a questionnaire,with demographic variables anddifferentscales to evaluate the degree of symptoms of depression, anxiety, insomnia, distress, and the level of work engagement(PHQ-9,GAD-7,ISI,IES-R,UWES-9).

**Results:**

Of the 217 participants, 46% were physicians, 42% were nurses and 12% were emergency medical technicians. We also found a femalepredominance of 66%, 55% were single and a total of 155 participants of whom 71% were frontline health workers. In our study, 54.8% of the HCWs had symptoms of depression, 68.2% had symptoms of anxiety and insomnia and 71.4% had symptoms of distress. Binarylogisticregressionanalysisshowedthat being married was associated with depression, anxiety, and insomnia, and being a frontlineworkerappeared to be a risk factor for depression and insomnia. Psychiatric support was an independentrisk factor for all psychiatric symptoms.In addition, living in a rural area was associated with depression, and age 31 or older was associated with anxiety. In addition, having a history of psychiatric illness was a risk factor for insomnia. Being a nurse was identified as a risk factor for psychiatric distress. We also found a moderatelevel of professional commitment to be a protective factor.

**Conclusions:**

Protecting healthcare workers is a crucial part of the public health response to the COVID-19 outbreak.

**Disclosure:**

No significant relationships.

